# Release of Indospicine from Contaminated Camel Meat following Cooking and Simulated Gastrointestinal Digestion: Implications for Human Consumption

**DOI:** 10.3390/toxins10090356

**Published:** 2018-09-03

**Authors:** Saira Sultan, Cindy Giles, Gabriele Netzel, Simone A. Osborne, Michael E. Netzel, Mary T. Fletcher

**Affiliations:** 1 Queensland Alliance for Agriculture and Food Innovation (QAAFI), The University of Queensland, Health and Food Sciences Precinct, Coopers Plains, QLD 4108, Australia; saira.sultan@uq.net.au (S.S.); g.netzel@uq.edu.au (G.N.); m.netzel@uq.edu.au (M.E.N.); 2 Department of Agriculture and Fisheries, Queensland Government, Health and Food Sciences Precinct, Coopers Plains, QLD 4108, Australia; cindy.giles@daf.qld.gov.au; 3 Commonwealth Scientific and Industrial Research Organisation, Agriculture and Food, St. Lucia, QLD 4067, Australia; simone.osborne@csiro.au

**Keywords:** indospicine, hepatotoxicity, meat, in vitro digestion, human

## Abstract

Indospicine, a hepatotoxic arginine analog, occurs in leguminous plants of the *Indigofera* genus and accumulates in the tissues of grazing animals that consume these plants. Furthermore, indospicine has caused toxicity in dogs following consumption of indospicine-contaminated meat; however, the potential impact on human health is unknown. The present study was designed to determine the effect of simulated human gastrointestinal digestion on the release and degradation of indospicine from contaminated camel meat following microwave cooking. Results showed no significant (*p* > 0.05) indospicine degradation during cooking or in vitro digestion. However, approximately 70% indospicine was released from the meat matrix into the liquid digesta during the gastric phase (in the presence of pepsin) and increased to >90% in the intestinal phase (with pancreatic enzymes). Following human consumption of contaminated meat, this soluble and more bioaccessible fraction of intact indospicine could be readily available for absorption by the small intestine, potentially circulating indospicine throughout the human body to tissues where it could accumulate and cause detrimental toxic effects.

## 1. Introduction

**Figure 1:**
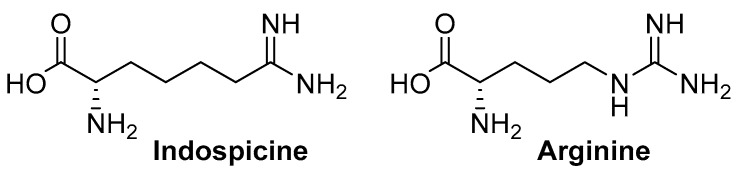
Chemical structures of the amino acids indospicine and arginine.

The non-proteinogenic amino acid indospicine (l-6-amidino-2-amino-hexanoic acid) is a hepatotoxic arginine analog (Figure 1) found widely in plants of the *Indigofera* genus [1]. This genus contains over 700 species distributed across tropical Africa, Asia, Australia, and North and South America, and includes species such as *I. spicata*, *I. hendecaphylla*, *I. linnaei*, *I. lespedezioides*, *I. vicioides* and *I. volkensii* that have all been reported to contain in excess of 500 mg indospicine/kg dry matter of foliage. Indospicine has been found to be directly toxic to livestock [2], and to also act as a secondary toxin due to its unusual ability to accumulate in tissues of livestock grazing on these plants [3,4,5].

Indospicine hepatotoxicity has been demonstrated in rats following a single dose of indospicine that inhibited protein synthesis and induced fatty changes in, and enlargement of, the liver [6]. Livestock consuming *Indigofera* develop similar indospicine-induced liver hepatotoxicity; however, symptoms of toxicity frequently become apparent only after extended periods of *Indigofera* consumption [7]. Before the toxicity of *Indigofera* to livestock was recognized in the 1950s, several *Indigofera* species (notably *I. spicata* and *I. hendacphylla*) were introduced as pasture legumes in the USA and Australia. As a result, these species are now widely found in tropical regions far beyond their native range, across Africa, Asia, Australia, the Americas, and islands of both the Pacific and Indian Oceans [2]. *I. linnaei* and *I. lespedezioides* are also regionally abundant with extensive native ranges in Australia and South America respectively [2]. *Indigofera* plants are palatable legumes that are readily consumed by livestock leading to the reported indospicine accumulation in the meat of cattle [3], camels [4], and horses [5,8].

Indospicine is non-proteinogenic and instead occurs in both plant and animal tissues as a free amino acid [3,4,5]. It is a competitive inhibitor of arginase [9] and DNA synthesis [10], and has been shown to cause liver degeneration [11] and abortion [12]. In fact, indospicine hepatotoxicity has been demonstrated in all animal species investigated to date with reports of acute and sub-chronic hepatotoxic evidence for rats, [13] mice, [14] rabbits, [15] guinea pigs, [7] sheep, [7] dogs [5], and cattle [7]. However, the severity of the toxicity appears to vary considerably between species with dogs being particularly vulnerable [16]. Indeed, secondary poisoning of dogs has been reported in dogs consuming meat naturally contaminated with indospicine arising from livestock that had grazed on *Indigofera* plants [5,17].

With respect to digestion of indospicine following consumption of *Indigofera* plants, previous in vitro rumen studies have shown that indospicine can be metabolized by the rumenal microbial system [18]. However, little is known about the stability, release, and potential degradation of indospicine within monogastric digestive systems, such as the human gastro-intestinal tract, in which a single-chambered stomach secretes enzymes and acid to facilitate digestion prior to passage into the small and large intestines. When considering the digestive stability of one component, it is important to consider not only the chemical structure of a compound but also the nature of its bond to the food matrix. Therefore, static and/or dynamic in vitro digestion models that mimic the human gastrointestinal digestion process are a common approach to determine the matrix release (bioaccessibility) and stability of food components (like nutrients and toxins) as an initial measure to predict their potential bioavailability [19,20,21,22].

In the present study, an in vitro model was utilized to investigate stability, release, and degradation of indospicine to better understand the potential human exposure following the consumption of indospicine-contaminated meat. Specifically, the aims of this research were to investigate the effect of cooking (microwave) on indospicine stability in camel meat naturally containing indospicine, and most importantly, predict the bioaccessibility and digestive fate of indospicine using a static in vitro digestion model mimicking the human gastric and small intestinal digestion process. To date and to the authors’ knowledge, this is the first study investigating the bioaccessibility of indospicine within a monogastric model system.

## 2. Results and Discussion

The hepatotoxic amino acid indospicine is found only in plant species of the *Indigofera* genus [1] and has been linked with poisoning of grazing livestock [2], and the deaths of dogs consuming contaminated meat from livestock that had grazed on these plants [5,17]. These canine deaths in Australia following the consumption of indospicine-contaminated camel [17] and horse meat [5] have raised both industry and consumer concern with regard to the potential contamination of meat for human consumption. The possible impact on human health is particularly concerning as all livestock grazing pastures that contain *Indigofera* plant species have the potential to accumulate indospicine as a free amino acid in their meat [3,4,23,24]. These indospicine residues are not readily excreted and can persist in tissues for up to 6 months after the cessation of *Indigofera* consumption, suggesting a strong affinity of indospicine with the meat matrix. Surveys of camel meat collected in Australia from animals killed in the field (in situ) and in abattoirs have demonstrated significant levels of indospicine residues above detectable limits (0.05 mg/kg), with meat from individual camels having levels up to 3.73 mg/kg [25]. Canine poisonings have occurred in the past following repeated consumption of indospicine-contaminated meat from the same or similar source repeatedly over several months [5,17]. The Australian supply of camel meat is sourced from more than 350,000 rangeland animals grazing arid inland regions of Australia [26], where *Indigofera* is also seasonally prevalent. These animals are slaughtered in abattoirs located within these regions before being supplied to growing niche markets in urban Australian Middle East and North African communities, as well as Central Australian local populations [26]. It is plausible that this lack of supply chain diversity could contribute to the same repeated exposure in these human consumers, and the potential health risk needs to be considered.

There are currently no identified studies providing epidemiological evidence or observational data indicating indospicine-induced adverse effects in humans. In the absence of such data, exposure risk can only be derived from available toxicity studies in dogs (often considered a model for human studies). The most substantial data available relates to thirteen different indospicine doses derived from two dog feeding experiments conducted for between 4 and 70 days [5,16]. A “lowest observed adverse effect level” (LOAEL) of 0.13 mg indospicine/kg bw/day is suggested based on observational data from the 70-day sub-chronic animal feeding experiment [5]. In this study, only minor histological liver lesions were observed in four dogs fed diets containing between 0.13 and 0.25 mg indospicine/kg bw/day [5]. A guidance value for human consumption of 1.3 µg indospicine/kg bw/day is thus proposed by dividing the selected LOAEL for dogs by an uncertainty factor of 10 to take into account mild degenerative changes to the liver in low dose dogs in the 70-day feeding trial, and by an additional factor of 10 to take into account intra species variation [27]. However, it can be postulated that a person with an average bodyweight of 70 kg [27] consuming the average Australian total daily meat intake of 143 g [28] could potentially consume 7.6 µg indospicine/kg bw/day if the dietary meat source was camel meat containing the reported 3.73 mg indospicine/kg [25]. This calculated intake of indospicine exceeds the proposed guidance value by a factor of 5. An additional factor of 10 could also be considered in the derived guidance value [27] due to the short duration of the literature study (70 days), and if this was implemented, then the calculated intake of indospicine could exceed the guidance value by a factor much higher than 5. Also, based on in vitro assessments of indospicine absorption using human intestinal cells [29], indospicine exhibits a 2-fold higher apparent permeability across an in vitro intestinal barrier compared to arginine (the amino acid analogue of indospicine). These findings indicate that indospicine is more readily absorbed than dietary arginine, suggesting preferential uptake that could potentiate further risks of toxicity.

Indospicine has been investigated in plant material by amino acid analyzer [5,30], high performance liquid chromatography (HPLC) with derivatization and UV detection [8,24] and liquid chromatography–tandem mass spectrometry (LC−MS/MS) [1,31]. However, the analysis of indospicine in meat is challenging due to low levels of contamination together with the complexity of the meat matrix. The incorporation of D_3_-l-indospicine as an internal standard in sample extracts as used in this study can be beneficial in LC−MS/MS analysis as it overcomes the matrix effects observed in previous studies [17].

**Figure 2:**
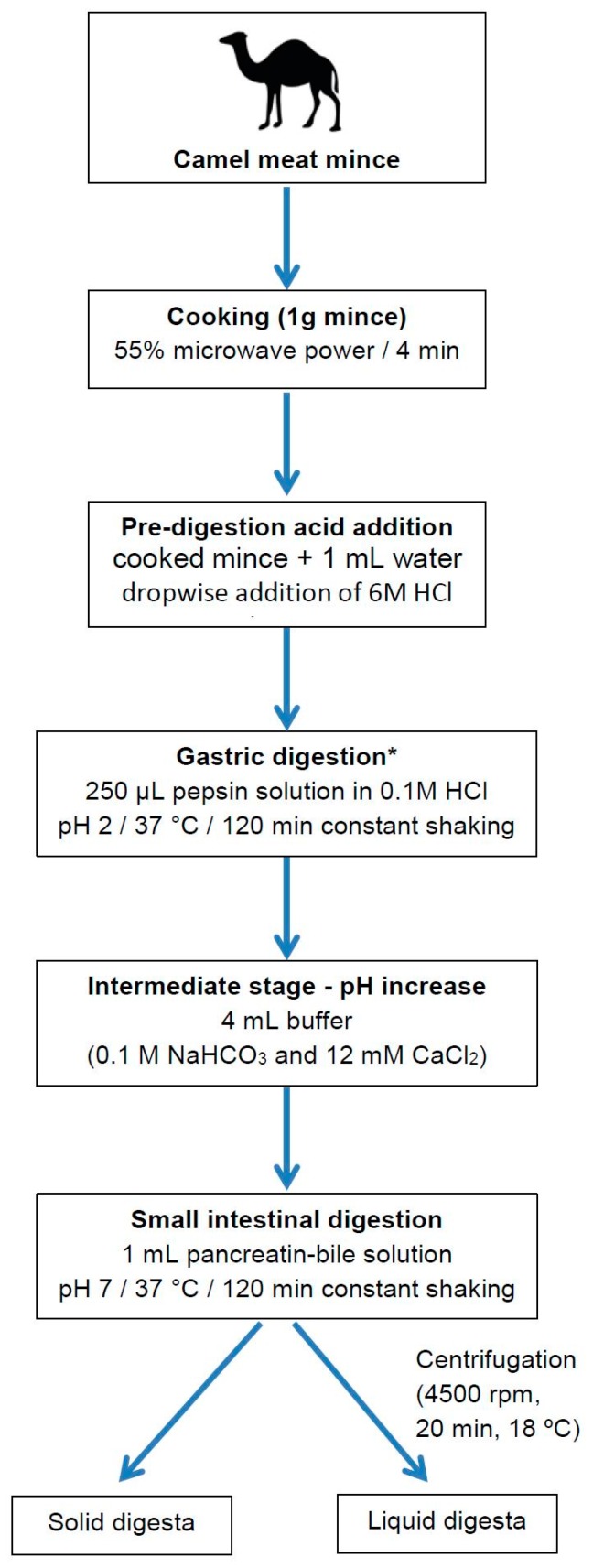
Schematic depiction of the stepwise in vitro digestion of camel mince under simulated human gastro-small intestinal conditions. (* Collection of gastric solid and liquid digesta as depicted for small intestinal digestion).

**Figure 3:**
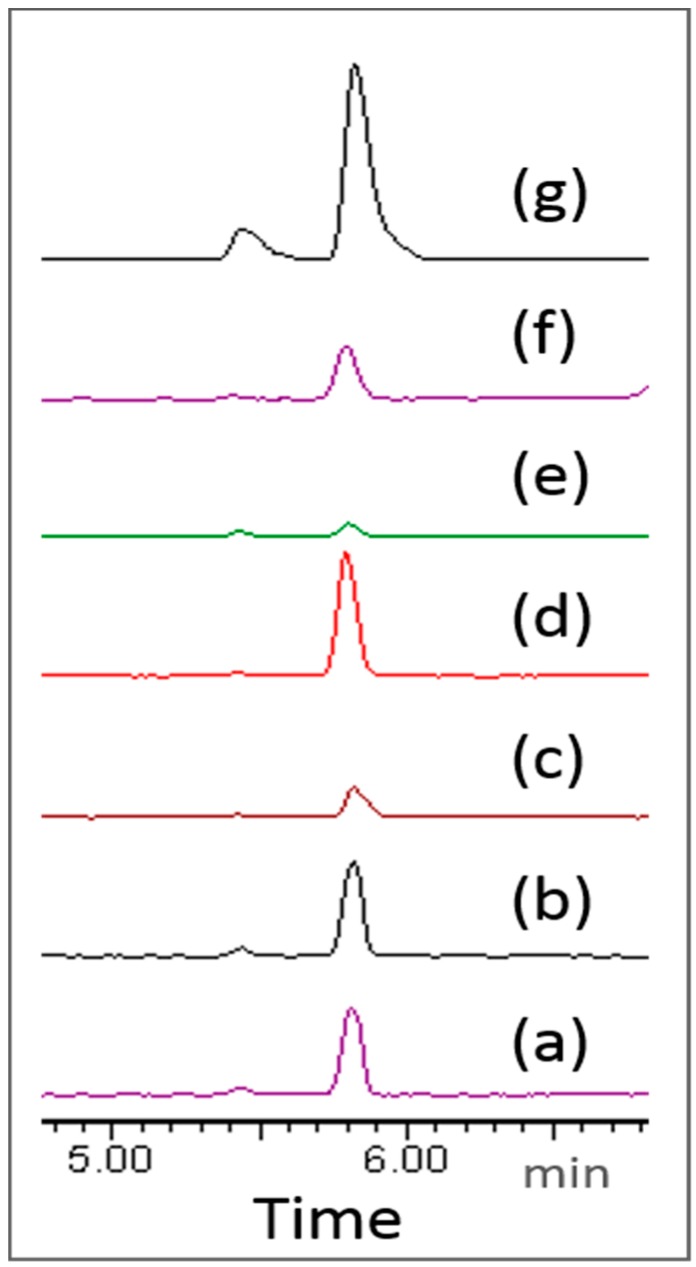
LC-MS/MS analysis of indospicine on Waters Micromass Quattro Premier triple quadrupole mass spectrometer utilizing a selected reaction monitoring (SRM) transitions of *m*/*z* 174 → 111 in (**a**) uncooked camel meat, (**b**) cooked camel meat, (**c**) solid phase of gastric digesta, (**d**) liquid phase of gastric digesta, (**e**) solid phase of small intestinal digesta, (**f**) liquid phase of small intestinal digesta, and (**g**) 0.05 mg/L standard indospicine solution.

**Figure 4:**
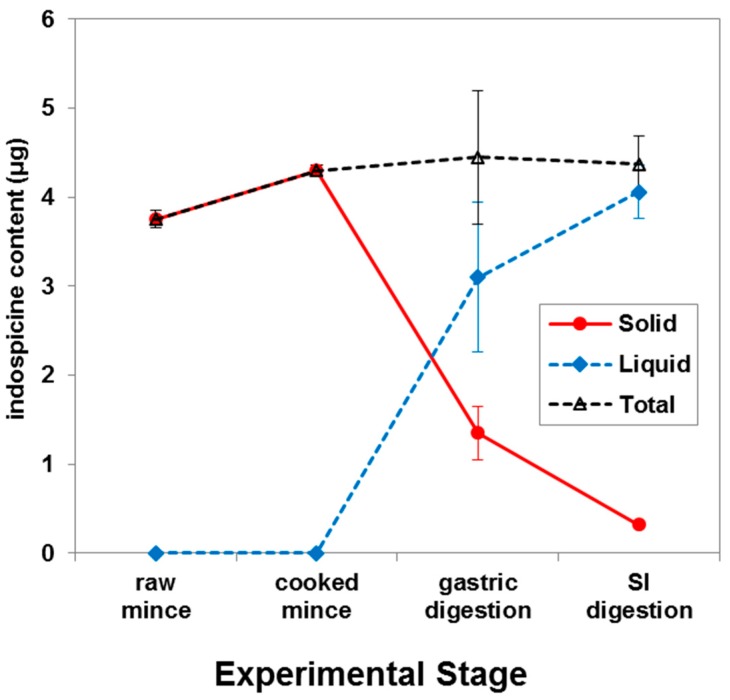
Indospicine content in raw, microwave cooked, and in vitro gastric and small intestinal (SI) digested indospicine-contaminated camel meat as determined by LC-MS/MS analysis. (All points are the mean of analysis of three replicates. Error bars show the SD).

In the present study, indospicine-contaminated camel meat (microwave cooked) was subjected to in vitro digestion through sequential addition of pepsin in 0.1 M HCl and pancreatin-bile solutions with an appropriate adjustment of pH to mimic human in vitro digestion (Figure 2). Liquid and solid digesta from the in vitro gastric and small intestinal digestion were separated by centrifugation, prior to determination of indospicine concentration by LC-MS/MS (Figure 3), utilizing the previously reported and validated method [32]. Indospicine concentration was also measured in uncooked and cooked camel meat using the same method. All studies were carried out in triplicate with results shown in Figure 4 and expressed as the mean and standard deviation (SD). To enable a comparison of the liquid and solid phases, results are presented as indospicine content (µg) in each phase rather than concentration.

Results from this study indicate that there were no significant changes (*p* > 0.05) in the total indospicine content, suggesting that indospicine was not degraded during microwave cooking or gastrointestinal digestion in vitro (Figure 4). Cooking causes shrinkage of collagen fibres [33] and also increases meat protein surface hydrophobicity [34]. Indospicine is a water-soluble free amino acid, and this meat matrix breakdown during cooking and subsequent in vitro digestion resulted in an almost complete release of this amino acid from the solid phase into the liquid phase (Figure 4). This is evident from the observed release of approximately 70% indospicine from solid to liquid phase after the incubation of cooked meat with pepsin (during the gastric phase). Moreover, digestion with pancreatin and bile in the small intestinal phase resulted in a total release of more than 90% indospicine into the liquid digesta.

In contrast to the observed lack of indospicine degradation in the present in vitro model of gastrointestinal digestion in a monogastric system, indospicine was almost 100% degraded when *Indigofera* plant material was incubated in camel foregut fluid for 48 h [18]. This differing result is indicative of the presence of microbes able to degrade indospicine in the camel gastric system. Indospicine was similarly degraded when incubated with bovine ruminal fluid [18], and further studies are underway to isolate the responsible microbes with the potential to be utilized as a preventive probiotic. However, the observed accumulation of indospicine in camel tissues suggest that even though indospicine can be degraded by foregut fermentation, complete degradation does not occur before passage of the digesta into the intestine and a significant portion of indospicine is then available for absorption [18].

It must be noted that in vitro digestion models have several limitations that should be considered when interpreting the results. For example, no current in vitro model is capable of replicating all aspects of in vivo digestion, absorption, distribution, biodegradation (including the metabolic activity of the gut microbiota), and elimination [35,36]. Nevertheless, our results indicate that indospicine is released from the meat matrix and appears resistant to human gastrointestinal conditions, potentially making it available for absorption in the small intestine from liquid digesta. Postprandial indospicine may circulate throughout the human body to tissues and organs, such as the liver, where it could accumulate over time and cause detrimental, toxic effects.

**Figure 5:**
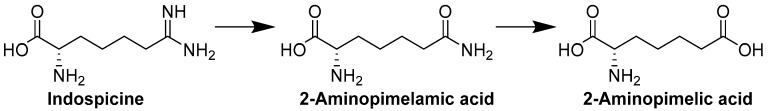
Hydrolysis of the amidino group of indospicine to corresponding amide (2-aminopimelamic acid) and acid (2-aminopimelic acid) under mild alkaline conditions as reported by Tan et al. [38].

There is no known mammalian enzyme that can degrade the amidino group of indospicine [37], and the preferred route to avoid indospicine toxicity is thus to prevent digestive uptake through degradation of the toxin during food processing. Our results suggest that the human digestive system does not have the capacity to degrade indospicine. Tan et al. [38] have recently reported that microwaving indospicine-contaminated camel meat under mild alkaline conditions (0.05% sodium bicarbonate, pH 8.8, 15 min) achieved 100% degradation of indospicine, with products identified as 2-aminopimelamic acid (major) and 2-aminopimelic acid (minor) (Figure 5). Such processing treatments may have ready applicability in the pet food industry given the recorded sensitivity of dogs to indospicine-contaminated meat, but are perhaps not appropriate in the processing of food for human consumption. Additionally, the metabolic fate and toxicity of indospicine hydrolysis products remains unknown and requires further investigation [38].

## 3. Conclusions

Simple cooking of contaminated camel meat in a microwave, as carried out in the present study, does not degrade indospicine. Moreover, in vitro human gastrointestinal digestion conditions also had no effect on indospicine degradation and only helped to release the toxin from the solid meat matrix into the liquid digesta. These observations imply that following human consumption and digestion of contaminated meat, indospicine could be readily bioaccessible for absorption across the small intestine. The toxicity of indospicine for humans is uncertain [39], but the known toxicity in dogs (often considered a model for human studies) is particularly concerning. Camel meat is not commonly consumed by the broader Australian population, but is eaten by local indigenous populations and some immigrant ethnic groups within Australia. Further risk assessments, particularly for these high exposure groups, need to be undertaken with additional consideration given to possible indospicine contamination of other red meat supply chains.

## 4. Materials and Methods

### 4.1. Reagents

Unless otherwise stated, all chemicals were purchased from Sigma-Aldrich (Castle Hill, Sydney, NSW, Australia), and were of analytical or HPLC grade. De-ionized water was used throughout all experiments.

### 4.2. Study Design

In this study, cooked indospicine-contaminated camel meat (both meat and juices) was subjected to in vitro digestion through the sequential addition of pepsin and pancreatin-bile solutions with an appropriate adjustment of pH as outlined in Figure 2 to simulate the human gastro-small intestinal digestion process. All samples were digested in triplicate for both gastric digestion alone and for gastric plus small intestinal digestion. Liquid and solid digesta after gastric and small intestinal digestion were separated by centrifugation, and the concentration of indospicine in the digesta (liquid and solid) and uncooked and cooked camel meat was measured by LC-MS/MS.

### 4.3. Camel Meat Samples

Camel meat samples were obtained from a previously described experimental feeding trial in which camels were fed a diet containing the pasture legume *Indigofera spicata* with consequential accumulation of indospicine in meat tissues [3,4]. This feeding trial was conducted under approval of the Animal Ethics Committee of the University of Queensland, QLD, Australia (AEC Approval No. SAFS/047/14/SLAI; Date of approval: 19 March 2014). Indospicine-contaminated camel meat samples collected at autopsy [4] were utilized in this present study. Camel meat samples were minced using a commercial meat mincer (PRO 1400 meat grinder, Kenwood, Prestons, NSW, Australia) to provide a homogenous sample and stored frozen at −20 °C until used for further analysis.

### 4.4. In Vitro Digestion of Camel Meat

The in vitro digestion of camel meat samples was performed following the method described by Netzel et al. [36] with some modifications (Figure 2). The gastric phase was 120 min to account for reported variations in gastric emptying following consumption of meals that produce larger particle sizes like meat [40,41]. A 120 min intestinal phase was also employed.

Prior to in vitro digestion, camel meat (minced, 1 g each) in 15 mL screw–cap Falcon tubes was cooked in a microwave oven (Panasonic Genius NN5752–750 Watts, Sydney, NSW, Australia) for 4 min at medium heat (55% power, approximate temperature 70 °C).

#### 4.4.1. Gastric Digestion

After cooking, samples were allowed to cool to room temperature and water (1 mL) was added to form a slurry. To prepare the samples for gastric digestion, the pH was lowered to 2.0 by the dropwise addition of HCl (6 M). To perform gastric digestion, 250 μL of pepsin solution (40 mg/mL pepsin from porcine gastric mucosa (1:2500 U/mg, Chem-Supply, Gillman, SA, Australia) dissolved in 0.1 M HCl) was added into the meat slurry and shaken manually to mix well. The mixture was incubated with continuous shaking at 37 °C for 120 min using an orbital mixer (RATEK Instruments, Boronia, VIC, Australia) placed in an incubator (Clayson IM550, Sydney, NSW, Australia). After 120 min constant shaking, tubes for gastric digestion were immediately centrifuged (4500 rpm, 20 min, 18 °C) to separate solid and liquid digesta.

#### 4.4.2. Small Intestine Digestion

The sample tubes identified for small intestinal digestion were processed further. To these tubes, 4 mL of buffer containing 0.1 M NaHCO_3_ and 12 mM CaCl_2_ was added dropwise to slowly raise the pH to 5.7. The digesta samples were mixed well and incubated for a further 30 min at 37 °C under constant shaking. This intermediate step was integrated to mimic the transition from the gastric to the small intestine environment. To start the small intestinal digestion, the pH of the mixture was further raised to 7.0 by the dropwise addition of 1 M NaOH followed by the addition of 1 mL of pancreatin-bile solution (8 mg/mL pancreatin from porcine pancreas (102557, USP Grade, MP Biomedicals, LLC, Illkirch, France) and 12 mg/mL porcine bile extract (B8631, Sigma-Aldrich, St. Louis, MO, USA) in 0.1 M NaHCO_3_). The digesta was again incubated at 37 °C for 120 min with constant shaking.

#### 4.4.3. Separation of Liquid and Solid Digesta

After completion of each digestion step (gastric and small intestinal) sample tubes were centrifuged with a Sigma 6K15 centrifuge (Sigma Centrifuges, Osterode, Germany; 4500 rpm, 20 min, 18 °C) to separate solid and liquid digesta. Liquid and solid digesta were stored separately at −40 °C until extracted and analyzed for indospicine.

### 4.5. Preparation of External and Internal Standards for LC-MS/MS Analysis

Indospicine analysis of all samples was conducted by LC-MS/MS utilizing synthetic indospicine as an external standard for preparation of a calibration curve and deuterium-labelled D_3_-l-indospicine as a stable isotopically labeled internal standard added to all samples and standard solutions to overcome matrix effects.

Synthesized indospicine as the external standard (>99% pure) and deuterium-labelled D_3_-l-indospicine (>99% pure) as the internal standard were kindly provided by Dr. Robert Lang and Prof. James De Voss, School of Chemistry and Molecular Biosciences, The University of Queensland, St. Lucia, QLD, Australia [42]. Stock solutions for both internal and external standards were prepared in de-ionized H_2_O with 0.1% heptafluorobutyric acid (HFBA) and were frozen at −20 °C until used. Internal (1 mg/L) and external (0.002–2 mg/L) standard solutions for indospicine LC-MS/MS quantification were prepared from the stock solutions and were stored frozen at −20 °C for no longer than a month before used.

### 4.6. Extraction of Indospicine from Camel Meat Samples and Digesta

Camel meat samples (uncooked, cooked, and both solid and liquid digesta) were extracted and analyzed by a previously validated and published liquid chromatography-tandem mass spectrometry method [32]. Prior to analysis, centrifugal filter units (Amicon^®^ Ultra 0.5 mL 3K, Merck, Bayswater, VIC, Australia) were pre-rinsed and centrifuged (Microcentrifuge 5424, Eppendorf, North Ryde, NSW, Australia) at 10,000 rpm for 20 min with de-ionized water (2 × 300 μL) to remove glycerine, then inverted and spun for 1 min at 1000 rpm.

Minced un-cooked camel meat was thawed, weighed (0.5 g), and mixed with 0.1% HFBA (25 mL), followed by homogenization (Polytron T25, Labtek, Brendale, QLD, Brendale, Australia) for 15 s. The homogenized samples were chilled (4 °C) for 20 min and then centrifuged at 4500 rpm for 20 min at 18 °C. Aliquots of 1.0 mL of the resulting supernatants were spiked with 100 µL internal standard (D_3_-l-indospicine, 1 mg/L in 0.1% HFBA), vortexed for 10 s, and a 450 μL portion was transferred into pre-rinsed centrifugal filters. The filtered sample mixture was then centrifuged (10,000 rpm, 20 min) and transferred to a limited volume insert (≈350 μL) for LC-MS/MS analysis.

Cooked meat and solid digesta were extracted and processed in a similar fashion to the raw meat. Liquid digesta (500 µL) was mixed with 0.1% HFBA (5 mL) and processed in a similar manner. All quantitations were calculated back to total indospicine content (µg) in either solid or liquid phase.

### 4.7. LC-MS/MS Analysis of Samples

Separation of the indospicine was achieved using a Waters ACQUITY UPLC^®^ system (Waters, Lane Cove, NSW, Australia) equipped with a Waters BEH C18 column (1.7 μm, 100 mm length, 2.1 mm i.d.) at 30 °C and a flow rate of 0.2 mL/ min. The mobile phase was a mixture of (A) H_2_O with 0.1% HFBA (*v*/*v*; pH 2.15) and (B) acetonitrile with 0.1% HFBA with the following gradient: 99% A to 70% A in 4 min, 70% A isocratic for 3 min, 70% A to 99% A in 1 min and 99% A for 2 min.

MS/MS detection was carried out using a Waters Micromass Quattro Premier triple quadrupole mass spectrometer with an electrospray ionisation (ESI) source operated in positive mode as previously described [32]. Eluted indospicine was quantitated utilizing selected reaction monitoring (SRM) transitions of *m*/*z* 174 → 111 (verified by transition of *m*/*z* 174 → 157) for indospicine, and *m*/*z* 177 → 114 (verified by transition of *m*/*z* 177 → 113) for D_3_-l-indospicine as internal standard. The capillary voltage was 2.79 kV; cone gas flow was 50 L/h; desolvation gas flow was 600 L/h. The source and desolvation temperatures were set at 150 °C and 350 °C, respectively. Argon gas collision energy of indospicine (15 and 12 eV) and D_3_-l-indospicine (15 and 15 eV) were set with cone voltage at 25 V.

### 4.8. Statistics

The data generated were processed using Microsoft Excel^®^ 2010 (Microsoft, Redmond, WA, USA). Statistical analysis was conducted using ANOVA (GraphPad Prism, Version 6, La Jolla, CA, USA) with a completely randomized design. Differences were considered significant when *p*-values were below 0.05.
